# Cross-Site Comparisons of Dryland Ecosystem Response to Climate Change in the US Long-Term Ecological Research Network

**DOI:** 10.1093/biosci/biab134

**Published:** 2022-08-16

**Authors:** Amy R Hudson, Debra P C Peters, John M Blair, Daniel L Childers, Peter T Doran, Kerrie Geil, Michael Gooseff, Katherine L Gross, Nick M Haddad, Melissa A Pastore, Jennifer A Rudgers, Osvaldo Sala, Eric W Seabloom, Gaius Shaver

**Affiliations:** Agricultural Research Service's Big Data Initiative and SCINet Program for Scientific Computing in Berwyn Heights, Maryland, United States; Agricultural Research Service's Big Data Initiative and SCINet Program for Scientific Computing in Berwyn Heights, Maryland, United States; US Department of Agriculture Agricultural Research Service's Jornada Experimental Range, Las Cruces, New Mexico, United States; New Mexico State University, Las Cruces, New Mexico, United States; Kansas State University, Manhattan, Kansas, United States; Arizona State University, Tempe, Arizona, United States; Louisiana State University, Baton Rouge, Louisiana, United States; Agricultural Research Service's Big Data Initiative and SCINet Program for Scientific Computing in Berwyn Heights, Maryland, United States; University of Colorado, Boulder, Colorado, United States; W. K. Kellogg Biological Station, Vermont, United States; Department of Plant Biology, Vermont, United States; W. K. Kellogg Biological Station, Vermont, United States; Department of Plant Biology, Vermont, United States; University of Vermont, Burlington, Vermont, United States; University of New Mexico, Albuquerque, New Mexico, United States; Arizona State University, Tempe, Arizona, United States; Global Drylands Center and the School of Life Sciences, Arizona State University, Tempe, Arizona, United States; University of Minnesota, St. Paul, Minnesota, United States; Marine Biological Laboratory, Woods Hole, Massachusetts, United States

**Keywords:** ANPP, disturbance, climate change, drought, LTER, wildfire

## Abstract

Long-term observations and experiments in diverse drylands reveal how ecosystems and services are responding to climate change. To develop generalities about climate change impacts at dryland sites, we compared broadscale patterns in climate and synthesized primary production responses among the eight terrestrial, nonforested sites of the United States Long-Term Ecological Research (US LTER) Network located in temperate (Southwest and Midwest) and polar (Arctic and Antarctic) regions. All sites experienced warming in recent decades, whereas drought varied regionally with multidecadal phases. Multiple years of wet or dry conditions had larger effects than single years on primary production. Droughts, floods, and wildfires altered resource availability and restructured plant communities, with greater impacts on primary production than warming alone. During severe regional droughts, air pollution from wildfire and dust events peaked. Studies at US LTER drylands over more than 40 years demonstrate reciprocal links and feedbacks among dryland ecosystems, climate-driven disturbance events, and climate change.

This Overview article synthesizes over 40 years of research on ecosystem response to climate change at diverse dryland sites. We define drylands as terrestrial, nonforested ecosystems potentially limited by water availability, where aboveground net primary productivity (ANPP) and precipitation are generally lower than at forested sites (Knapp and Smith 2001). To mark the 40th anniversary of the US National Science Foundation's Long Term Ecological Research (US LTER) Network, this article is one of five that overview and address responses of forest and freshwater, coastal, and marine pelagic ecosystems to climate change highlighting US LTER research.

Drylands in the US LTER Network provide the long-term and broadscale perspective needed to understand ongoing responses to climate change in ecosystems that cover over a third of the Earth's land surface and are projected to expand with global warming (Huang et al. 2016, IPCC 2021). Drylands are found on every continent and have adapted to different climate regimes. Drylands can differ in key ecosystem properties, such as plant species composition, primary production, and carbon and nitrogen cycling, that affect their response to climate and disturbance (Shachak et al. 2004, Haversustad et al. 2006, Maestre et al. 2012, Delgado-Baquerizo et al. 2013, Hobbie and Kling 2014, Berdugo et al. 2020, Maestre et al. 2021). The primary limiting factor to dryland productivity likely varies among and within these systems (e.g., temperature versus rainfall versus nitrogen) at seasonal, interannual, or multidecadal time scales. Plant communities adapted to different climate regimes may therefore not respond uniformly to changing climate and climate-driven disturbances. Comparing the patterns in climate and ecological responses of different dryland ecosystems to past climate events can provide important insights to predicting future dynamics (e.g., Heisler-White et al. 2009).

Comparisons of ecosystem responses across US LTER dryland sites can also provide insight into climate change effects on ecosystem state transitions, feedbacks, and the global carbon cycle. Drylands are often near state-transition boundaries such that localized extreme climatic events or a climate-driven disturbance event can lead to state transitions, such as from grasslands to shrublands, that are challenging to reverse (Suding et al. 2004, Bestelmeyer et al. 2011, Fukami and Nakajima 2011, Gilbert et al. 2020, Seabloom et al. 2020, Collins et al. 2021) but can be buffered by a biologically diverse plant community (Isbell et al. 2015). These state changes can also have broader scale feedbacks to climate via effects on region-scale energy and water cycles (Beltrán-Przekurat et al. 2008) and the global carbon cycle (e.g., Ahlström et al. 2015). Even without considering state transitions, global drylands typically have higher turnover rates of carbon pools compared with other terrestrial systems and are important in driving year-to-year variability in the global carbon cycle (figure 1; Ahlström et al. 2015), likely a reflection of precipitation variability and its effects on semiarid dryland production.

**Figure 1.**
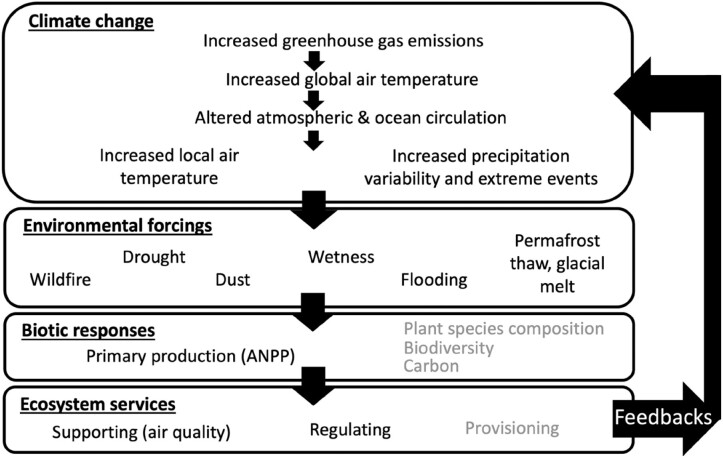
Dryland systems overview of key drivers, ecosystem responses, services, and feedbacks. Anthropogenic warming interacts with natural variability to drive climate change across the globe. Broadscale climate drivers and human driven land use (e.g., prescribed burns or tilled versus untilled agricultural systems) then interact with the landscape to create local changes in temperature and precipitation, which contribute to wildfire, dust storms, and flooding events further dependent on the landscape. We highlight in this article the ecosystem response of primary production, specifically aboveground net primary production (ANPP), with side stories into how ANPP response coincides with plant species composition. Ecosystem services are supporting, regulating, and provisioning. Some processes, such as air quality and carbon sequestration then feedback to local to regional energy and water budgets and global CO2 concentrations.

Disturbance events at dryland sites, such as wildfires, floods, and dust storms, are the result of broadscale climate patterns and dynamics interacting with the landscape and can have a large negative impact, as was shown in studies at US LTER sites (figure 1; Stanish et al. 2012, Abraha et al. 2015, Ratajczak et al. 2019, Collins et al. 2020, Peters et al. 2020, Collins et al. 2021). The frequency and intensity of landscape-scale disturbance events are projected to increase with global warming (IPCC 2021) while being region (USGCRP 2018) and site dependent. Although conceptual frameworks have been developed (e.g., Peters et al. 2011, Gaiser et al. 2020), comparing responses with similar disturbance events across US LTER sites has not been a focus of previous studies. This study fills this gap by relating landscape-scale disturbances to plot-level data on ecosystem responses collected at diverse dryland US LTER sites.

A comparison of ecosystem responses across the eight US LTER dryland sites can also leverage information from both long-term observations and experiments. Observations allow consideration of initial state conditions and existing ranges of variability across a large spatial and temporal extent (Shaver et al. 2000). Experiments can determine causality and can explore the potential impact of projected climates at fine spatiotemporal scales. Pairing observational data with plot-level experiments leads to stronger understanding of ecosystem responses to environmental perturbations. Some observational studies have documented divergent responses of dryland systems to large-scale climate variability or drought, for example. Those studies suggest differential sensitivity of carbon cycling or other processes (e.g., Petrie et al. 2016) but require mechanistic explanations that come from manipulative experiments.

The US LTER dryland sites include programs at three midwestern US sites (Konza Prairie, KNZ; Cedar Creek Ecosystem Reserve, CDR; Kellogg Biological Station, KBS), three southwestern US sites (Central Arizona–Phoenix, CAP; Jornada Basin, JRN; Sevilleta, SEV), and two polar sites (figure 2; Arctic, ARC; Antarctic McMurdo Dry Valleys, MCM). These eight sites represent a diverse suite of drylands distributed across the continental United States and at both poles (figure 2b). Sites from the continental United States represent temperate grasslands (Cedar Creek, Minnesota; Konza, Kansas), agricultural landscapes (Kellogg, Michigan), warm to hot temperate deserts (Sonoran Desert: Phoenix, Arizona; Chihuahuan Desert: Jornada, New Mexico) or their ecotone with temperate grasslands (Sevilleta, New Mexico). Polar sites are cold deserts in the Arctic (Toolik Lake, Alaska) and Antarctic (MCM). Collectively these eight sites cover the diversity of land cover types in global drylands (Maestre et al. 2021).

**Figure 2.**
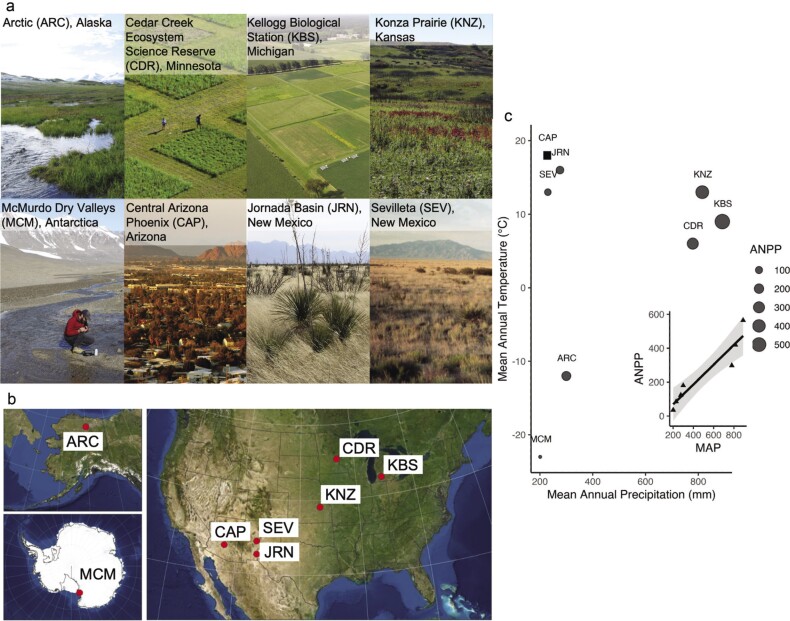
The geographic location, productivity, and climate of eight dryland US LTER sites. (a) Site-based photographs. (b) Site locations within the United States and each pole. (c) Site-level mean annual precipitation (MAP; x-axis) ranges from 200 to 900 millimeters per year, mean annual temperature (MAT; y-axis) ranges from –23 to 18 degrees Celsius, and long-term mean annual aboveground net primary productivity (ANPP, indicated by circle size) ranges from 35 to 565 grams per square meter per year across sites. CAP does not have ANPP measures and is indicated by a square. Photographs: The site-based photographs were obtained from https://lternet.edu/site-image-galleries; the ARC and CAP CC BY-SA 4.0., JRN E Zambello/LTER-NCO CC BY 4.0, CDR photo by Jacob Miller, and KNZ photo by Jill Haukos.

The eight dryland US LTER sites fall into three clusters based on their mean annual precipitation and temperature values (figure 2c). Hot deserts and grasslands in the Southwest United States (CAP, JRN, SEV) have low mean annual precipitation (MAP), high mean annual temperature (MAT), and moderate ANPP, whereas temperate grasslands and agricultural sites in the US Midwest (KNZ, CDR, KBS) have higher MAP, high MAT, and relatively high ANPP (figure 2c). Polar sites (ARC, MCM) are considered cold deserts with low (MAP) and (MAT) and corresponding low or zero mean ANPP (figure 2c). Precipitation (rain, snow) inputs are the source of available water at temperate sites, whereas glacial melt (MCM) or the freezing and thawing of saturated soils and the permafrost (ARC, MCM) creates available water at polar sites. The positive correlation between ANPP and MAP reflects the importance of precipitation inputs to production at temperate sites (figure 2c insert).

In the present article, we compare broadscale patterns in climate and synthesize dryland ecosystem responses across eight diverse dryland sites in the US LTER using long-term observational and experimental data and literature. We present standardized climate data across sites, reference literature discussing likely climate projections, and weave in the climate and disturbance event focus of dryland US LTER studies. For each climate driver, we organize ecosystem response by region (Midwest, Southwest, Arctic, Antarctic) reflecting site clusters in figure 2c, and the wide range of primary resource limitations in diverse dryland ecosystems. We focus our synthesis on primary production and plant species composition. Primary production by plants is an ultimate and only source of energy for food webs and is critical for sequestration of carbon from the atmosphere (O'Connor et al. 2017). Plant species composition can help explain changes in primary production while also being a proxy for key ecosystem services (Grman et al. 2010, Tilman et al. 2014, Gross et al. 2015). Primary production and plant species data are collected across the US LTER Network, including all the sites in this synthesis (Hobbie et al. 2003). We then present a case study of dryland climate interacting with dryland ecosystems to influence the ecosystem service of air quality. Finally, we identify key and diverse ways in which dryland response can feed back to regional and global climate.

## Supporting data

We compared broadscale patterns in climate across all sites using standardized data sets of temperature and drought, which provide comparable data across sites for time scales exceeding the US LTER program. Growing seasons, seasonal climate, and their impact on ecosystems differ among sites; to generalize climate impacts across sites, we annually averaged these climate data. Monthly minimum and maximum temperatures were annually averaged for each site from the Berkeley Earth 1-degree gridded product (http://berkeleyearth.org/archive/data; Rohde et al. 2013). Anomalies were computed as 1.5 standard deviations from the 1930 to 2019 average. We used drought data from annual averages of the monthly (SPEI-1 m) and 3-month (SPEI-3 m) Standardized Precipitation–Evapotranspiration Index (SPEIbase v2.6; https://spei.csic.es/database.html; Vicente-Serrano et al. 2010), based on 0.5-degree gridded CRU TS v4.03 observational data for the period 1930 to 2018. In SPEI, dry conditions are defined as when precipitation inputs are less than potential evapotranspiration (PET), with PET calculated using the FAO Penman–Monteith equation (Beguería et al. 2014). Therefore, SPEI is influenced by both precipitation and temperature but is constrained by the modeled resistance of vapor flow through stomata, leaf area, and the soil surface, such that this drought index is particularly relevant for ecological applications (e.g., Rudgers et al. 2018). SPEIbase is not available at MCM. Only a few temperate sites have calculated SPEI based on local weather-station data, with PET calculated differently, and so we do not include site-level SPEI and primary production comparisons in this article.

We leveraged continent-scale observations of wildfires and air quality to broaden the regional implications of drought on drylands. We used data on lightning and human-caused fires recorded by the National Interagency Fire Center from 2001 to 2020 in the US Southwest (www.nifc.gov/sites/default/files/document-media/Lightning-Human-Caused_2.pdf). The Southwest geographic area is defined by the National Interagency Coordination Center and Fire Center as the states of Arizona and New Mexico and the Federal lands within Texas and Oklahoma west of the 100th meridian. We calculated the number of hectares burned per fire event from 2001 to 2020 in the US Southwest. We used data on particulate matter in air obtained from the Environmental Protection Agency (www.epa.gov/air-trends/air-quality-cities-and-counties) at cities near temperate sites for 1990–2019. Particulate matter concentrations are important indicators of air quality, a key ecosystem service in drylands, and typically increase with more wildfire (PM10) and dust events (Eagar et al. 2017). Finer particles (PM2.5) pose a greater health risk because they can penetrate further into the lungs (Eagar et al. 2017). We differentiated particles with diameter less than or equal to 2.5 (PM2.5) and 10 (PM10) microns. Annual PM10 values are the second highest 24-hour concentration measured at the site during each calendar year, whereas PM2.5 values are the 98th percentile of 24-hour averaged concentrations averaged over a 3-year period.

Site-level precipitation data were also obtained to directly compare precipitation with ANPP over time at the five temperate dryland sites (KBS, KNZ, CDR, SEV, JRN; table 1). For production at each of the five sites, annual estimates were calculated by averaging plot-level values from multiple locations. Colocated growing season or annual precipitation amount was obtained by aggregating daily data (table 1). Precipitation is not a major indicator of water availability at polar sites (ARC and MCM), and the urban site CAP does not have an equivalent ANPP data set, and so these three sites were excluded from this visualization.

**Table 1.** Data sources for aboveground net primary productivity (ANPP) in figure 2 and ANPP and precipitation (PPT) in figure 3.

Site	ANPP location and method of aggregating	ANPP data package	PPT location and method of aggregating	PPT data package
Arctic	Mean (*M*) = 180 grams [g] per [m^2^] per year, 1982–2015. The total ANPP by all functional types at historic tussock tundra site; sampling across four large blocks with four to eight replicate samples per block (replication varied among years)	knb-lter-arc.10004.8 (Shaver et al. 2017)	–	–
Cedar Creek Ecosystem Reserve	*M* = 298 g per m^2^ per year; estimates were calculated by averaging across sox control plots that were unplowed and unfertilized in field circa 2012,2013, 2016, and 2017 were missing from this data set and estimated using values from a nearby plot	knb-lter-cdr.14.9 (Tilman 2020)	Daily precipitation from composite of on-site stations (Cedar Weather Station, CC1, and CC2) summed for each year.	knb-lter-cdr.419.9 (Seeley 2020)
Jornada Basin	*M* = 123 g per m^2^ per year, 1990–2018, was estimated by averaging total production at three grassland locations (BASN, IBPE, SUMM)	knb-lter-jrn.210011003.104 (Peters and Huenneke 2020)	Daily precipitation collected at each location was summed for each year, and averaged across locations	knb-lter-jrn.210425001.75 (Yao et al. 2020)
Kellogg Biological Station	*M* = 564 g per m^2^ per year, 1990–2018, was estimated by averaging biomass over six 1-ha replicates with five stations/plot sampled every year at a successional herbaceous control plot managed through annual fire	knb-lter-kbs.19.85 (Robertson and Snapp 2020)	Same location as ANPP	knb-lter-kbs.17.27 (Robertson 2019)
Konza Prairie	*M* = 419 g per m^2^ per year, 1984–2018, was calculated by averaging total biomass from an upland site (WS001d; soil type, fl) that is annually burned and ungrazed	knb-lter-knz.55.13 (Blair and Nippert 2020)	Daily data collected at KNZ Headquarters was summed for each year	knb-lter-knz.14.17 (Nippert 2021)
McMurdo Dry Valleys	*M* = 35 g per m^2^ per year, 1990–2011, was based on the summed average ash free dry mass of high and low abundance orange mats (orange mats are most extensive) and experimental studies of how long it takes for mats to regrow on scraped rocks (about 4 years)	personal communication with Diane McKnight (Kohler et al. 2015)	–	–
Sevilleta	*M* = 98 g per m^2^ per year, 1999–2019, was estimated by averaging black grama grassland fall biomass across 30 spatially distributed sample plots of 1 m2	knb-lter-sev.319.2 (Hallmark et al. 2019, Rudgers et al. 2019)	Growing season precipitation was collected from the nearest meteorological station and spring (November-May) and fall (May–October) amounts were summed for each year	knb-lter-sev.319.2 (Hallmark et al. 2019, Rudgers et al. 2019)

*Note:* For figure 3, total ANPP and PPT annual estimates were found. CAP is an urban site and did not have comparable ANPP estimates.

## Patterns in temperature

**Figure 3.**
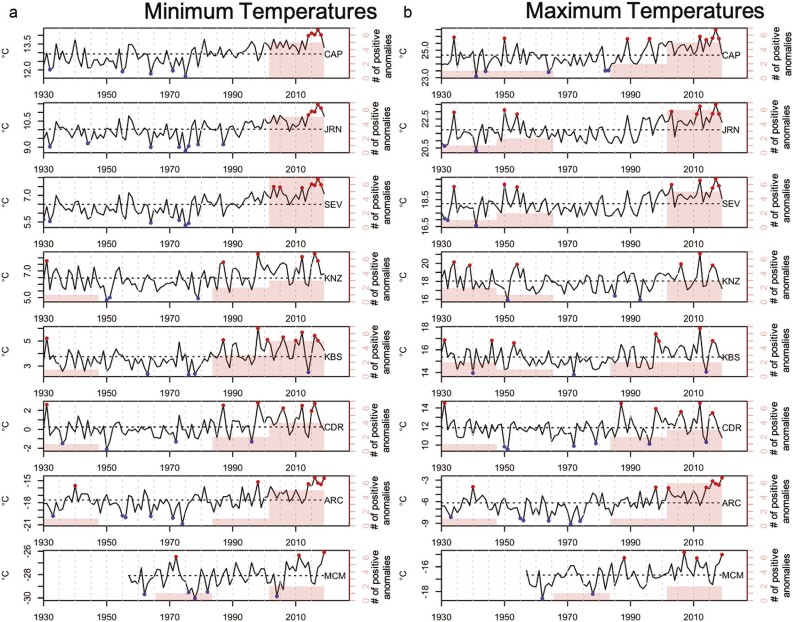
Annual average minimum and maximum temperature for the region surrounding each site. The sites are organized by mean annual temperature, with the warmest at the top and the coldest at the bottom. The top three are US Southwest sites, the middle three are US Midwest sites, and the bottom two are Arctic and Antarctic sites. (a) Minimum and (b) maximum temperatures. The dots indicate values that are 1.5 times the standard deviation above (red points) and below (blue points) the long-term mean (dashed line) for each site. The red histogram (right axis) designates the number of positive temperature anomalies per period for five periods of 18 years each.

Warming has occurred at all sites from either 1982 to 2019 or 2000 to 2019, based on coarsely gridded data that show an increase in the number of positive minimum and maximum temperature anomalies compared with previous decades (figure 3). These results sometimes differ from temperature observations at the site-level for individual US LTER sites that typically have not shown warming trends through time (e.g., JRN, Peters et al. 2012; ARC, Hobbie et al. 2017). This discrepancy between fine and coarse observations is due in part to the larger variability in site-based observations influenced by topography or other local features and, in some cases, to the shorter period available to calculate means (e.g., ARC, MCM). Gridded observational products aggregate site-level observations within and between coarse one-degree cells. Most US LTER sites have multiple meteorological stations distributed across the landscape to capture this finer scale variability in climate that is smoothed in the coarser gridded product (Greenland et al. 2003, Peters et al. 2021).

## Ecological responses to temperature

Direct effects of temperature are covered in this section, and the combination of changes in temperature and precipitation are covered in the section on drought below. In general, many ANPP responses to temperature vary by dryland region and depend on species composition (Epstein et al. 1997, Klemm et al. 2020).

In the Midwest, warming experiments at CDR suggest that elevated temperatures increase ANPP, diversity, and carbon storage (Cowles et al. 2016, Reich et al. 2020). Increases in plant diversity can buffer the potential negative effect of warmer temperatures in lowering soil moisture on ANPP (Cowles et al. 2016) or promote diverse soil microbial communities and enzyme activities that modify ANPP (Steinauer et al. 2015). In C4-dominated grasslands, warming can advance emergence and canopy green up of the dominant grasses and reduce ANPP of subdominant forbs (Fay et al. 2011). Warmer winter temperatures also lead to less precipitation falling as snow (Robertson and Hamilton 2015), which serves as an insulator to protect the soil surface from dramatic temperature swings. Lower amounts of snow increase the frequency of freeze–thaw cycles that break up soil macroaggregates and limit productivity in systems with minimal winter vegetation cover, such as croplands (Ruan and Robertson 2017) or sites where the vegetation and litter has been removed before winter by fire (Pastore et al. 2021).

In the Southwest, warming stresses plants that are already experiencing high growing-season temperatures (Peters et al. 2011). For example, warm autumns preceded low tree seed production for dominant species at SEV (Parmenter et al. 2018), which both lowers recruitment potential and removes valuable food sources from the ecosystem. Surpluses in water and availability of nutrients, such as nitrogen, limit the negative effects of warming on ANPP and plant community structure (Collins et al. 2017). Therefore, warmer nighttime temperatures are not as detrimental to grassland ANPP as warmer daytimes might be because there is added moisture loss in the daytime from plant transpiration (Collins et al. 2017).

In the Arctic, warmer temperatures over the past 30 years led to increases in plant cover (especially woody shrubs; Hobbie et al. 2017), and expanding habitat that is suitable for long-lived plants, such as sedges (McGraw et al. 2015). Increased air temperature reduces soil moisture through increased evaporation from the thin surface soil layer, but these effects are counteracted in lowland areas by the accumulation of runoff from the thawing of permafrost at upland sites (Hobbie et al. 2017).

In the Antarctic, warmer temperatures generally increase glacial melt, which feeds into ephemeral streams to resuscitate microbial mats and initiate primary production (Sohm et al. 2020). Cooler temperatures in earlier decades (1986–2001; Doran et al. 2002) resulted in drought conditions that lowered stream flows, reducing total microbial mat biomass in streams, but increasing mat biodiversity (Kohler et al. 2015) as habitat heterogeneity increased. Streamflow variability also influenced the relative abundance of diatoms and diatom diversity in microbial mats (Stanish et al. 2012). Local pockets of soil moisture from snow melt promote the increased abundance and distribution of nondominant soil fauna (Andruizzi et al. 2018) but have a negligible impact on primary production. For phytoplankton in the adjacent lake, warmer temperatures led to thinner lake ice and increased light availability. When coupled with nutrient influx from seasonal streamflow, aquatic ANPP increased (Bowman et al. 2016).

## Patterns in precipitation

Both amount and variability in precipitation are important to dryland ANPP (Gherardi and Sala 2019) and are likely to continue to change with global warming (IPCC 2021). Warmer air can hold more water, increasing precipitation event sizes across all sites (IPCC 2021). Annual average precipitation has increased over the Midwest and is likely to continue to increase in the Midwest and Alaska, with the largest increases in winter and spring precipitation (USGCRP 2018). In the Southwest, however, climate model projections of changes in precipitation are more uncertain, with some suggesting no change in precipitation outside of historic variability (e.g., Gutzler and Robbins 2011), whereas others project drying (Greene and Seager 2016). Many US LTER sites, including the temperate dryland sites in this synthesis, examine precipitation effects on primary production through complementary manipulations and observations. Both observational and experimental data can be used to examine legacy effects of extreme amounts of precipitation on ANPP (Sala et al. 2012).

## Ecological responses to changes in precipitation

**Figure 4.**
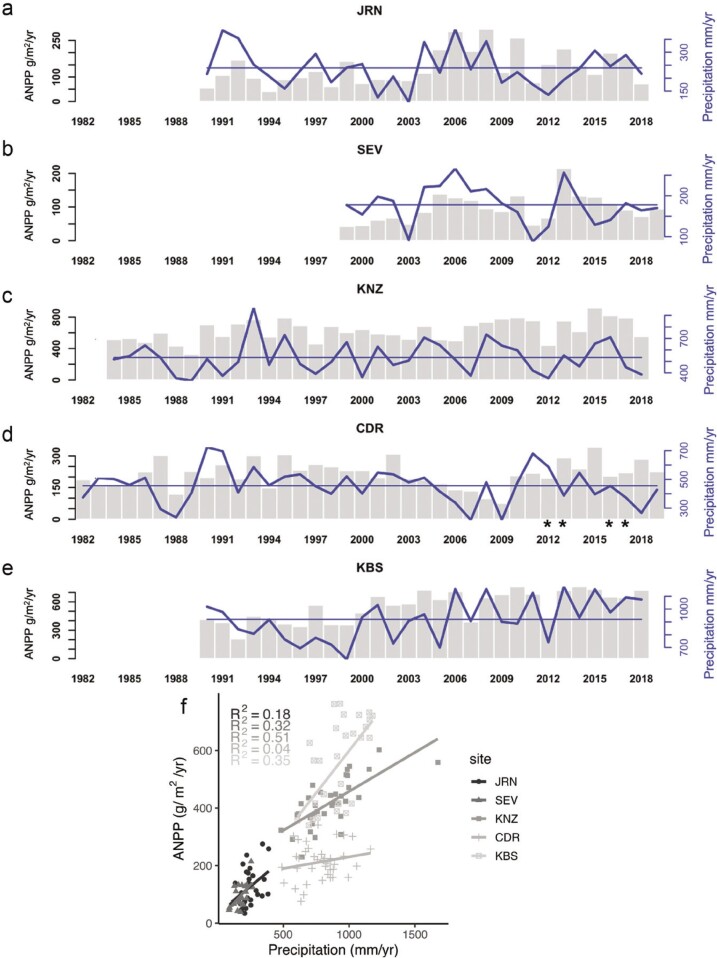
Site-based observations of dryland production and corresponding total annual precipitation. Total aboveground production (in grams per square meter per year) in grey vertical bars, and precipitation (in millimeters per year) in blue lines. Horizontal blue line designates the average precipitation over the period shown. The asterisk (*) under the ANPP bars at CDR designates estimates of ANPP that were derived from a nearby plot. (a) JRN, (b) SEV, (c) KNZ, (d) CDR, and (e) KBS. Increased annual precipitation generally corresponds to larger primary production across temperate sites (f).

Long-term data collected from US LTER sites have shown that observed plot-scale ANPP is positively related to precipitation (figure 4; Lauenroth and Sala 1992, Huxman et al. 2004), but the strength of the correlation varies among dryland sites (figure 4f). Beyond a linear effect, multiple years of high or low precipitation and changes in precipitation variability can lead to legacy, lagged, and oscillating effects on ANPP (e.g., Haddad et al. 2002, Gherardi and Sala 2015b). The large amount of variability in ANPP can often be better explained by separating the long-term data into wet or dry periods (Peters et al., 2012, 2021).

In the Midwest, in natural grasslands and experimental controls at CDR, low precipitation in 1987–1988 caused initial reductions in ANPP, followed by a decade long oscillation with above and below average ANPP (figure 4d; Haddad et al. 2002). Precipitation and ANPP at CDR were significantly correlated from 1987–2012 (r = .48, *p* < .05), but after 2012, production and precipitation were decoupled, producing a weak correlation for the entire period (figure 4f). Peak production in 2015 corresponded with slightly lower than average precipitation (figure 4d), and warmer temperatures (figure 3), perhaps indicating that this system is becoming less water limited. The effects of low precipitation on production can be amplified when paired with conventional tillage treatments (Robertson et al. 2014), whereas fields exposed to less intense continuous no-till treatments were more resilient to drought.

Intra-annual precipitation variability experiments at KNZ and KBS have characterized plot-level responses to fewer but larger precipitation events (e.g., Knapp et al. 2002, Heisler-White et al. 2009, Wilcox et al. 2015, Jones et al. 2016, Hess et al. 2018, Felton et al. 2019) and potential shifts in the seasonal timing of rainfall and drought (e.g., Denton et al. 2017). Increased time between rainfall events typically lowered ANPP (Knapp et al. 2002, Wilcox et al. 2015) and plant species diversity through increased variability in soil moisture (Knapp et al. 2002). Reductions in plant productivity were even larger when the increased time between rainfall events was paired with reduced rainfall (Knapp et al. 2002). Sensitivity of above- and belowground production to timing and event size of precipitation can differ between (Heisler-White et al. 2009) and within (Wilcox et al. 2015) different grassland types, suggesting the importance of community composition in ecosystem response to precipitation variability. Community structure can be resilient to long-term exposure to fewer but larger precipitation events; it took 10 years before forb cover and richness increased significantly in response to a 15-year precipitation manipulation (Jones et al. 2016). At KNZ, historical precipitation variability was smaller than projected variability incorporated into climate change experiments, and this created differences in ANPP responses in observational and experimental studies. Nippert and colleagues (2006) found that grassland ANPP was positively related to growing season precipitation variability as observed over the 1984–1999 study period; however, short-term experiments that imposed twice the observed precipitation variability identified a negative response in ANPP.

**Figure 5.**
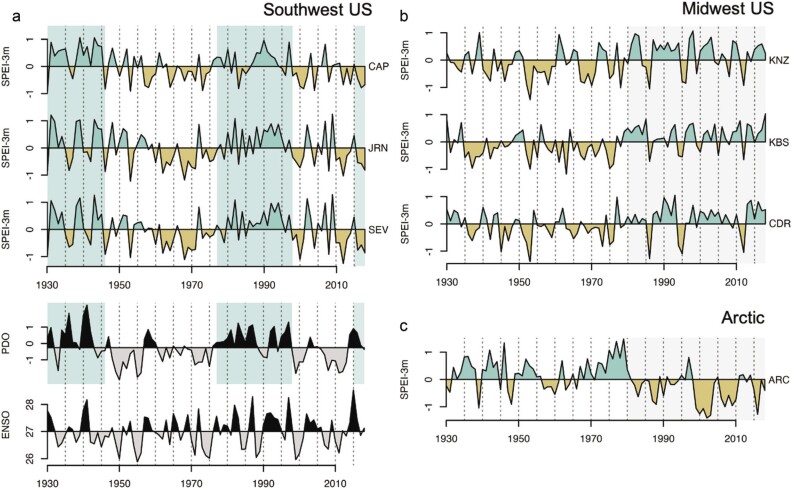
Long-term patterns in drought (SPEI) at dryland sites where data were available (MCM excluded). Annual averages of monthly SPEI, where positive values (teal) designate wetter conditions (precipitation is greater than potential evapotranspiration; P > PET), whereas negative values (brown) designate drought conditions (P < PET). (a) Southwestern sites (CAP, JRN, SEV) and modes of climate variability: the Pacific Decadal Oscillation (PDO) index (www.ncdc.noaa.gov/teleconnections/pdo, Mantua et al. 1997), and the El Niño Southern Oscillation (ENSO) index (Nino 3.4; https://psl.noaa.gov/gcos_wgsp/Timeseries/Nino34, Rayner et al. 2003). Teal shading indicates the positive phase of the PDO, which generally coincides with wetter conditions at Southwest sites. (b) Midwestern sites (KNZ, KBS, CDR), and (c) Arctic site (ARC). Grey shading from 1980 to the present indicate the beginning of the US LTER program and the 1980 s regime shift.

Even though the Midwest sites have been experiencing relatively wetter conditions since the 1980 s (figure 5b), there are relatively few studies that examine the effects of a net increase in precipitation on ecosystems. Indeed, increased moisture throughout the soil profile may be playing a role in the increase of woody vegetation across tallgrass prairie landscapes (Briggs et al. 2005), but changes in land management, especially fire suppression practices, which began in the 1980 s, may also be contributing factors (Ratajczak et al. 2014). KNZ, CDR, and KBS have been conducting long-term and ongoing supplemental water addition factorial experiments (started in 1992,2007, and 2000, respectively; Collins et al. 2012, Knapp et al. 2012, Robertson and Hamilton 2015, Wilcox et al. 2016, Yang et al. 2018, Kimmel et al. 2019). As would be expected in these water-limited systems, mesic grasslands irrigated to reduce growing season water stress (mean of approximately 30% more water than ambient conditions) generated almost 50% more ANPP over a 20-year period (Collins et al. 2012, Knapp et al. 2012, Wilcox et al. 2016), with accompanying increases in soil carbon dioxide (CO2) efflux (Knapp et al. 1998) and long-term changes in soil physical properties (Caplan et al. 2019) but few other detected changes in biogeochemical cycling (Wilcox et al. 2016) or species richness (Collins et al. 2012). Initially, increases in ANPP averaged around 40%, but in the second decade of this long-term experiment, community restructuring of the dominant C4 grass caused a much larger increase (64%) in ANPP in irrigated plots (Knapp et al. 2012). Studies pairing irrigation with fertilization have identified even larger increases in ANPP (Robertson and Hamilton 2015, Yang et al. 2018) dependent on treatment intensity and increasing declines in species richness and other functional diversity indicators over time (Kimmel et al. 2019). These results from US LTER studies have large implications for how wetter baseline conditions paired with management can affect crop and biofuel production as well as carbon and nutrient cycling as fertilizer leaches from the system and root and soil carbon storage flux (Robertson and Hamilton 2015, Yang et al. 2018). New irrigation experiments are now focusing on whether multiple years of water addition have altered the sensitivity of these grasslands to interannual climate variability or to experimentally imposed droughts. KBS has recently installed a drought experiment, and treatments are likely to expand to include increased precipitation and continue to increase understanding of these climate effects.

In the Southwest, JRN and SEV (and the broader Chihuahuan Desert) experienced multiple years of wetter conditions from 2004 to 2008 and higher grassland production than expected based on long-term trends (figure 4a, 4b; Peters et al. 2014, Rudgers et al. 2019). In degraded shrublands where grasses occur infrequently, a sequence of wet years can initiate grass establishment and survival processes that maintain grasses without management inputs and potentially lead to a future state change reversal from shrubland back to grassland (Peters et al., 2012, 2014). Observations at JRN show that not all wet periods have the same impact on the production of perennial C4 grasses in degraded shrublands (Peters et al. 2021). Wet periods with similar amounts of rainfall every year (1984 to 1988) did not lead to increases in C4 production; rainfall manipulations at SEV produced a similar response (Báez et al. 2013). Meanwhile, a wet period (2004–2008) with large pulses of rainfall (2006,2008) resulted in unusually large increases in grass production, where the number of consecutive wet years was a better predictor of ANPP than precipitation amount (Peters et al. 2021). Years with large pulses of rainfall are expected to become more frequent in the future, suggesting that perennial grass production may also increase, and that past relationships between ANPP and rainfall may not be indicative of the future.

Similar dryland sites (receiving less than 300 millimeters of annual precipitation) in a global study generally increased ANPP with increased interannual precipitation variability (Gherardi and Sala 2019). These ANPP responses can be dependent on ecosystem and community composition. At JRN, increased interannual variability in precipitation typically led to decreases in grass cover and increases in shrub cover (Gherardi and Sala 2015a), resulting in similar total ANPP levels (Gherardi and Sala 2015b). Rainfall manipulations at SEV determined that a single large precipitation event during the summer monsoon season had a larger positive impact on ANPP than smaller events spread out over the growing season due to the response of the dominant C4 grass (Thomey et al. 2011).

In the Arctic, significant changes in the seasonality of precipitation and in the proportion of precipitation that falls as rain versus snow are being observed (e.g., Jones and Driscoll 2022, this issue), with varying implications for soil moisture and plant production (Cherry et al. 2014). At the plot scale, research in Scandinavian tundra, similar to the tundra at Toolik Lake, has shown no clear relationship between annual variation in precipitation and productivity, and multiyear manipulations of precipitation have had marginal to negligible effects on productivity (Press et al. 1998, Dormann and Woodin 2002). Nevertheless, at the landscape-scale, vegetation patterns and productivity are strongly related to soil moisture, soil water movement, and associated nutrient availability (e.g., Chapin et al. 1988).

In the Antarctic, snow collects in patches across the landscape, but these patches are typically small in total water volume and their contribution to hydrologic processes is very localized for a short period (Obryk et al. 2020). Stream algal biomass is not influenced by snow levels. The few large snow patches (order of 100 square meters) melt and create water to supply fluvial-like features (aka water tracks) that move water downslope in the subsurface rather than at the surface.

## Drought

Long-term data such as that gathered in US LTER experiments and observational studies are essential to understand the immediate and persistent effects of drought. Temperature and precipitation interact to influence drought, as is represented by SPEI (figure 5). Warmer temperatures interacting with precipitation events drive changes in water available to plants. Warmer air temperatures also increase evaporative demand, which decreases the amount of water available to plants in the soil profile, both through reductions in infiltration and losses of small precipitation events from reaching the land surface.

### Patterns in drought

Recent decades were relatively drier in the Southwest (CAP, JRN, SEV; figure 5a), wetter in the Midwest (KNZ, KBS, CDR; figure 5b), and drier in the Arctic (figure 5c). These multidecadal patterns were often broken up by one or more dry or wet years across regions. Patterns in the drought index also indicate a difference in the frequency and duration of drought across regions (Reid et al. 2016, USGCRP 2018, IPCC 2021, Ducklow et al. 2022, this issue). In the Southwest, drought has historically recurred on average every 25 years and corresponds with the multidecadal phases of the Pacific Decadal Oscillation (PDO; Chylek et al. 2013) punctuated by the interannual variability of the El Niño Southern Oscillation (ENSO; figure 5a; Williams et al. 2014, USGCRP 2018). Warmer temperatures may cause Southwest sites to break this cyclicity, or cause aridity thresholds to be crossed, with impacts on dryland processes including ANPP (Berdugo et al. 2020). The Midwest transitioned from a more-than-50-year period of drier conditions to wetter conditions after 1980, whereas the Arctic transitioned to drier conditions in the same time period (figure 5b, 5c). The 1980 s climate regime shift has been documented globally in climate and ecosystem response and was shown to be linked to anthropogenic warming combined with the volcanic eruption of El Chichón in the tropics (Reid et al. 2016). Ecological results from US LTER sites are most appropriate for the climatic period of occurrence (wet or dry) and may not reflect responses in the alternative climatic period (dry or wet). Data from even the longest running US LTER sites may be insufficient to capture multidecadal climate cycles and transitions (Cusser et al. 2021), emphasizing the importance of coupling observations with experiments in considering ecosystem response. Although the Midwest sites have experienced relatively wetter conditions in recent decades, there have been droughts lasting one or more years within these decades and projections for increases in growing season droughts. Therefore, we highlight below the studies that have examined drought impacts on ecosystems.

### Ecological responses to drought

In the Midwest, interactive effects of warming and rainfall on plot-scale plant production varies by species (Pastore et al. 2020) and cannot be predicted from their effects in isolation (Reich et al. 2020). Experimental warming, reduced rainfall, nitrogen fertilization, and elevated CO2 in combination actually increased plot-scale ANPP by 43% at CDR, whereas the expected effect based on responses to each driver alone was a 75% reduction (Reich et al. 2020). A 2-year drought experiment and mid-summer heat wave found that drylands are resistant to heat waves, but extreme drought caused ANPP to drop below levels observed over a 30-year period (Hoover et al. 2014). However, the following year, ANPP returned to average levels as the community restructured. Dryland production is more sensitive to fewer and larger rainfall events during periods of drought (Felton et al. 2019). In addition, the seasonality of ecosystem exposure to drought may be important. Reduction in spring rainfall did not reduce ANPP, whereas summer rainfall reduction significantly lowered ANPP (Denton et al. 2017).

In the Southwest, growing season drought has become more variable in the northern extent of the Chihuahuan Desert since the 1980 s (Rudgers et al. 2018). Aridity variance and average values can drive ANPP, with relationships dependent on ecosystem characteristics (Rudgers et al. 2018). A 5-year experimental drought followed by 4 years of water addition (similar to the 2004–2008 period; figure 4), found that multiyear drought decreased dominant C4 grass production, and following wet periods only slightly increased grass production; shrub production was resistant to both rainfall reduction and addition (Báez et al. 2013).

In the Arctic, there is little evidence that drought alone is sufficient to significantly reduce production except in small, deeply thawed, and very well-drained soils (Oberbauer and Dawson 1992). Major summer droughts occur, but their main impact is to dry out the soil surface and increase the chances of wildfire (e.g., in 2007). In plot-scale manipulation experiments, reduced precipitation had little or no impact relative to manipulations of temperature, light, and (especially) nutrient availability over 5 years (Press et al. 1998, Dormann and Woodin 2002). However, observations indicate that interannual and spatial variations in greenness may be related to temperature and drought (Hobbie et al. 2017).

### Wildfire interactions with drought

**Figure 6.**
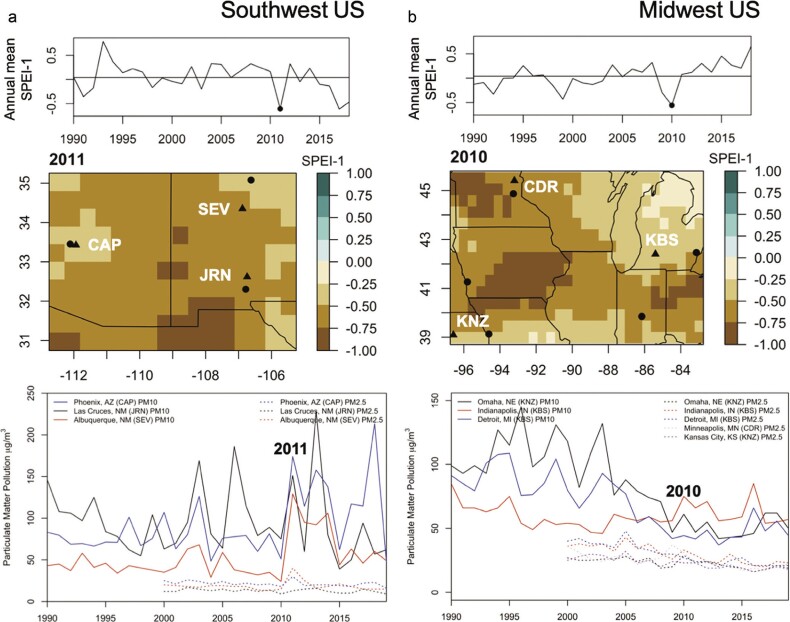
Comparisons of drought and air quality for Southwest and Midwest regions. (a) Southwest (31 N to 35 N and –113E to –107E) and (b) Midwest (39 N to 46 N and –97E to –82E) regions. (top) A time series from 1990–2019 of annual SPEI 1-month anomalies averaged within regions. (middle) The SPEI values are color coded for the 2011 drought in Southwest and 2010 drought in Midwest; US LTER sites are shown as triangles, and nearby cities where air quality was recorded are shown as black points. (bottom) The atmospheric concentration (in micrograms per cubic meter) of coarse particulate matter (PM10, solid lines) and fine particulate matter (PM2.5, dashed lines) pollution at cities designated in middle panel.

**Figure 7.**
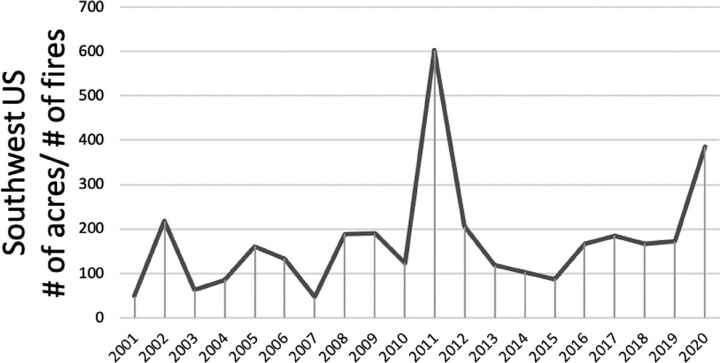
Wildland burned in the Southwest United States over the past 20 years. The total number of hectares burned was divided by the number of fires for each year from 2001–2020 in the Southwest United States. Fires include both lightning and human-caused fires. The Southwest geographic area is defined by the National Interagency Coordination Center and Fire Center as the states of Arizona and New Mexico and federal lands within Texas and Oklahoma west of the 100th meridian. The 2011 peak in hectares per fire event corresponds with the 2011 regional drought for the southwest and regional peaks in low air quality (figure 6a).

In many drylands, fire is a historically natural and frequent disturbance, but frequency, extent, and severity are sensitive to climate. Drier surface conditions (figure 5a, 5c) are associated with longer wildfire seasons and more frequent and larger wildfires at US LTER sites in the western United States and Alaska (Mack et al. 2011, Rocha et al. 2012, USGCRP 2018). Region-wide drought conditions in 2011 in the southwest preceded by fuel accumulation after a wet period (figure 6a) coincided with the largest area of land burned by wildfire in Arizona and New Mexico over the period from 2001 to 2019 (figure 7; Williams et al. 2014).

In the Midwest, prescribed burns used to prevent shrub encroachment and mimic historic wildfire regimes can increase short-term energy, water, and nitrogen availability. For systems in which there is another limiting factor of growth (e.g., nitrogen at CDR and KBS), fire does not alter long-term ANPP or plant species composition (e.g., Knops 2006, Dickson and Gross 2013). But for grassland systems subject to shifting relative resource availability and multiple resource constraints such as the tallgrass mesic prairie at KNZ, fire removes litter and alters the energy environment, which leads to increased cover and dominance of warm-season grasses, reduced cover of forbs and cool-season grasses, reduced overall species diversity, and increased total ANPP (Blair 1997). In a watershed-scale experiment at KNZ, a subset of long-term plant sampling plots experiencing a natural drought (high temperatures and low precipitation in the growing season) were subject to a growing season wildfire in 2011 (Ratajczak et al. 2019). Plots that experienced only drought showed no decreases in plant diversity or change in community composition. However, plots that experienced both drought and wildfire experienced large reductions in grass cover and large increases in forb cover, with an overall increase in biodiversity. Two years later, plant community structure still had not returned to preburned levels (Ratajczak et al. 2019), suggesting strong and persistent impacts of wildfire and drought.

In the Southwest, wildfire remains a frequent driver of ecosystem dynamics for landscapes with sufficient fuel (Gosz and Gosz 1996, Wang et al. 2019). At JRN, wildfire was important historically (before 1900 s) when semicontinuous grass and herbaceous cover permitted fire spread, but wildfire became infrequent after much of the landscape transitioned to woody shrubs separated by bare soil or sparse grass cover (Drewa et al. 2001). Low-intensity prescribed burns can lower soil water content under shrubs and enhance grass cover (Wang et al. 2019). At SEV, a wildfire that occurred during a long-term nighttime warming, nitrogen addition and increased precipitation experiment reduced the dominant grass cover by 75%, which resulted in an increase in forb species richness and biomass over the subsequent 4 years (Collins et al. 2017). Wildfire and climate variability (PDO) caused nonlinear and lagged correlations between production and precipitation and changed the long-term reordering of dominant grass species in this system (Collins et al. 2020).

**Figure 8.**
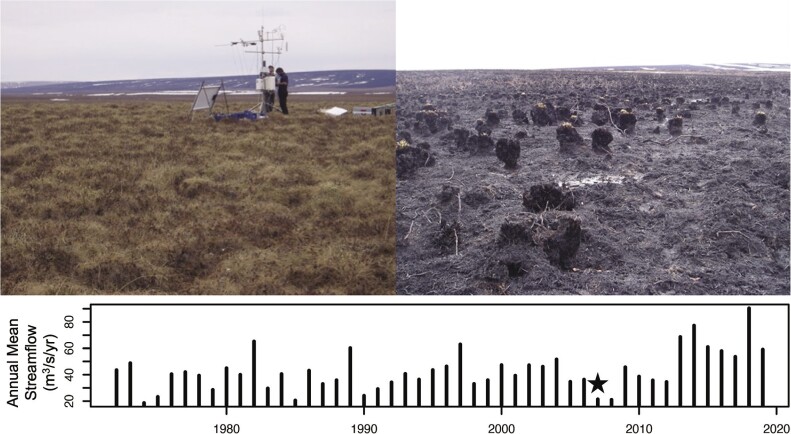
Arctic tundra landscape response to wildfire and corresponding low streamflow in 2007. Photographs taken in late May of 2008 at the control site (left) looking north toward the severely burned site (right) after the 2007 Anaktuvuk River Burn. This fire burned over 1039 square kilometers on the north slope of Alaska from late July to early October 2007 when it was finally extinguished by cooler temperatures and the first snowfall of the year. Annual mean streamflow (in cubic meters per second per year) at the US Geological Survey gauge Kuparuk River near Deadhorse was low in 2007 and 2008, indicating corresponding drought conditions.

In the Arctic tundra, fire is a rare disturbance (Rocha et al. 2012) that is increasing in frequency (USGCRP 2018). Warmer temperatures at ARC in recent decades (figure 3) have supported increases in fire fuel load via increased shrub growth (Hobbie et al. 2017), whereas drought in 2007 (figure 5c), indicated by drastically low streamflow (figure 8) coincided with a large wildfire event in 2007 (figure 8) that caused losses of approximately 50 years of accumulated carbon and approximately 400 years of nitrogen (Mack et al. 2011). Remarkably, 5 years after this fire, carbon sequestration doubled at burned compared with unburned sites (Jiang et al. 2017). Although vegetation growth was initially limited by the loss of canopy, postfire vegetation recovery was rapid, promoted by residual nutrients in organic matter from the fire and by nutrients released from the thawing permafrost (Boelman et al. 2011, Mack et al. 2011, Rocha and Shaver 2011). Despite this rapid vegetation response, thawed soils remain deeper and nutrient export was higher in burned areas 10 years after the fire, indicating persistent long-term losses of stored carbon and nutrients (Abbott et al. 2021). Recent research suggests that the incidence of Arctic lightning ignitions is projected to increase with projected increased precipitation for the region (Chen et al. 2021). The negative feedback of precipitation on drought adds to the complexity of future fire dynamics in the Arctic.

## Links between drought, wildfire, and dust disturbances, dryland response and a key ecosystem service: Air quality

Climate drivers, in particular drought, can interact with land surface properties to cause major impacts on ecosystem services, as has been demonstrated at US LTER sites. Wildfires and dust storms can reduce air quality, especially in combination with drought (Kandakji et al. 2020), but effects depend on how the climate system (e.g., temperature, precipitation, wind speed) interacts with the land surface (e.g., topography, soil properties, traits of the plant species). For example, drought can harm the plants and cyanobacteria to increase dust emissions. Growing season drought decreases grass cover, which further increases dust flux (Alvarez et al. 2012), removing soil carbon and nitrogen from bare soil. State transitions to shrublands and the associated increase in bare soil enhance dust flux (Webb et al. 2021). Experimental drought altered the community of cyanobacteria, which build biological soil crusts that stabilize the soil (Fernandes et al. 2018).

In the present article, we examined the relationships between drought and airborne particulate matter (PM) from wildfires and dust storms, based on work from US LTER sites. Following Duniway and colleagues (2019), we differentiated between dust emissions, defined as particulate matter (PM2.5, PM10) in the atmosphere and wind movement of soil particles, defined as the local redistribution of surface soil material by wind. Dust emissions and wind erosion are linearly related, and increase during drought (Gillette and Passi 1988, Houser and Nickling 2001).

Large and fine particulate matter pollution peaked during the driest years in both the Southwest and Midwest (figure 6). On average, concentrations of large particles were higher in the Southwest (maximum 225 micrograms [μg] per cubic meter [m3]) with increasing variability since 2000 (figure 6a) in contrast to with Midwestern cities (maximum 150 *μ*g per m3) with decreasing variability since 2010 (figure 6b). Peaks in PM10 concentrations across the Southwest (figure 6a) coincide with the peak in the number of hectares per wildfire event in 2011 in the Southwest (figure 7). Other years with locally high PM10 emissions, such as Phoenix in 2018, were likely related to local land use changes, such as increased soil tilling (figure 6a; Van Pelt et al. 2017). By contrast, fine particle concentrations for both regions show little change over time (figure 6).

**Figure 9.**
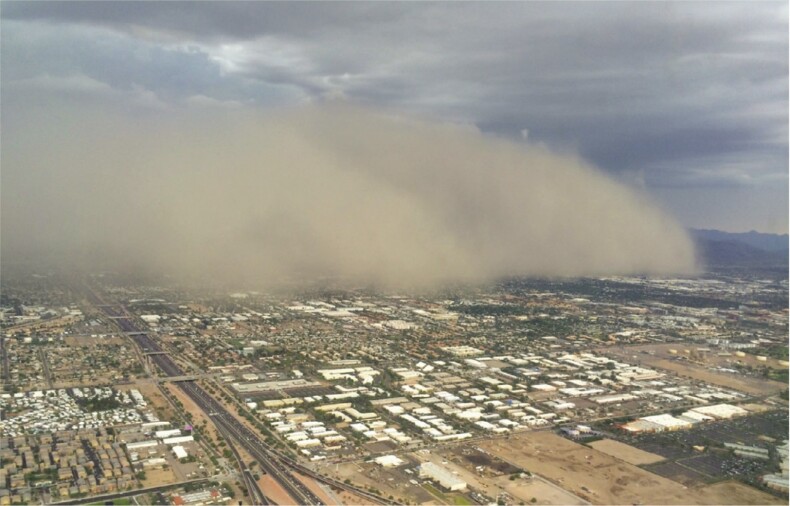
Dust storm (haboob) over CAP in 2015. CAP is in Tempe, Arizona. Photograph: Eagar and colleagues (2017), taken by A. Anbar.

In the Southwest, high winds during the summer monsoon season combined with dry soils and bare ground in fallow agricultural fields can create haboob dust storms (figure 9; Eagar et al. 2017). Downdrafts from a convective thunderstorm, typical of summer monsoon periods, can push a wall of dust (a haboob, a term originating in Arabic) ahead of the storm, increasing PM10 and PM2.5 concentrations by orders of magnitude. At CAP, haboob frequency ranged from 3 to 20 per year (20 haboobs in 2011) over the period 2005–2014, with higher frequency in drier years, but reduced frequency in hyper arid years when precipitation was less than 100 millimeters such as in 2009 (Eagar et al. 2017) and 2020.

## Flooding

Warmer temperatures are expected to drive more frequent and intense flooding events in dryland systems that negatively affect primary production, but the mechanisms vary across regions. In the Midwest, floods typically are saturation-excess events resulting from high rainfall rates on saturated or frozen ground. In the Southwest, flash floods often result from high-intensity precipitation, low infiltration rates of desert soils, and rapid runoff on steep slopes. Impermeable surfaces exacerbate flooding in urban areas. In polar drylands, floods result from permafrost thaw events and glacial melt. Therefore, increased precipitation intensity and warming can both contribute to enhanced flooding in dryland systems.

In the Midwest, croplands studied at KBS, seasonal inundation can have more deleterious effects than drought in reducing yield. For example, although drought during the growing season could reduce plant productivity, irrigated fields prevent this (Robertson and Hamilton 2015). However, early season inundation prevents crop planting at appropriate times or can wash out seeds after planting, with fewer management options. Intense precipitation events in the spring can decrease soil water content at shallow depths and limit water availability later in the growing season (Hess et al. 2018). These events coupled with soil disturbance (e.g., tilled systems) lead to an increase in soil water that leaves the system via runoff or evaporation compared with undisturbed systems (Hess et al. 2020), leaching away key nutrients from this system to downslope systems (e.g., coastal systems; Reed et al. 2021, this issue).

In the Southwest, multiyear positive precipitation anomalies correspond to generally more productivity in drylands but flooding events can modulate that relationship and lead to plant anoxia or produce a competitive advantage for species that can tolerate anaerobic conditions (e.g., Peters et al. 2006).

**Figure 10.**
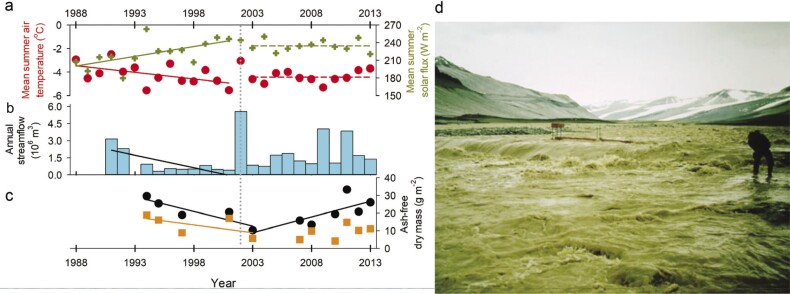
Antarctic stream biomass response to warming-induced flooding event in 2001–2002. The vertical dashed line at 2001–2002 in panels (a), (b), and (c) designates the large melt event. In 2001–2002, mean summer (DJF) air temperature and incoming solar radiation peaked (a) and caused the largest annual volumes of stream flow observed on nine streams in the Lake Fryxell basin over the entire period of reference (1988–2013) (b). (c) Stream cyanobacterial mat ash-free dry mass (average ash free dry mass in grams per square meter) of orange (Phormidium-dominated) and black (Nostoc-dominated) mats from permanent transects on four streams in the Lake Fryxell basin were lower the year following the large melt event. Stream biomass had been decreasing over the decade leading up to 2001–2002, corresponding with cooler conditions at the site. Black mat biomass recovered 10 years after the flooding event in 2001, even though climate drivers (a) were stationary. Panels (a)–(c) are revised from Gooseff and colleagues (2017).(d) Photograph: C. Lynch of the upstream view of the 1986–87 January flooding, Figure 81 in Chinn and Mason (2016).

In the Antarctic, more frequent warming events and increased surface energy inputs are associated with enhanced glacial melt and increased flooding (figure 10; Bergstrom et al. 2020). In 2001–2002, La Niña and a positive southern annular mode (SAM) phase led to a heat wave (figure 10a) and extreme glacial melt, which caused severe flooding at MCM (figure 10b; Fountain et al. 2016) scouring microbial mats (Kohler et al. 2015) and delivering suspended sediment to downstream lakes, where increased turbidity reduced light and primary production by phytoplankton (Foreman et al. 2004). Biomass of microbial mats and lake phytoplankton recovered within a few years (figure 10c; Fountain et al. 2016, Gooseff et al. 2017).

## Dryland feedbacks to climate

Understanding dryland landscape feedbacks to climate is crucial for prioritizing local land management strategies (e.g., Jenerette et al. 2016) and reducing uncertainty around the global land carbon sink (e.g., Ahlström et al. 2015). Long-term research from US LTER that relate ANPP to climate provide insights into how dryland ecosystems influence the global carbon budget (e.g., Petrie et al. 2016, Reich et al. 2020).

In the Midwest, experimental warming and increased rainfall that led to increases in total NPP (above and belowground NPP) at CDR corresponded with relatively smaller increases in soil respiration, and therefore increased total ecosystem carbon pools (Reich et al. 2020). Climate drivers of plant biodiversity are particularly important in considering feedbacks, because increases in plant biodiversity can significantly increase soil carbon stocks (Pastore et al. 2021). Over twice as much carbon is found in roots than in aboveground tissues at CDR's long-term experiment BioCON (Pastore et al. 2021), highlighting the need for future analyses to explore whether belowground responses match aboveground responses to climate change and how that may affect total ecosystem soil carbon storage.

Land–atmosphere coupling amplified the intensity of the summer flash drought in 2012 over the Great Plains, when a warm spring created an earlier and longer demand for soil moisture by plants, which then limited evaporative cooling and exacerbated warming in summer months (figure 5b; Wolf et al. 2016). Most ecosystems in the Great Plains experienced dramatically reduced net ecosystem production during this summer as water became severely limited.

Less intense management of row-crop agriculture, including land conversation to perennial bioenergy crops or conservation areas at KBS, increases carbon sequestration and reduces nitrous oxide emissions (e.g., Robertson et al., 2000, Gelfand et al. 2013, 2017).

In the Southwest, the transition from grasslands to shrublands altered the albedo and the latent and sensible heat of the near-surface system (Beltrán-Przekurat et al. 2008). The relatively darker shrubland canopy decreased albedo, and the deeper-rooted shrubs were able to access more groundwater and increased latent heat flux. Sensible heat effects were more dependent on shrub type because of differences in canopy height and root structure (Beltrán-Przekurat et al. 2008). In contrast, irrigation of vegetation in urban environments, such as at CAP (Phoenix, Arizona), promotes evaporative cooling, with potential increases in production when plants are not stressed (Jenerette et al. 2016).

In the Arctic, the wildfire of 2007 released a pulse of CO2 to the atmosphere from the combustion and decomposition of previously frozen soil organic matter (Mack et al. 2011, Rocha and Shaver 2011, Jiang et al. 2017, Abbott et al. 2021). The ongoing press of warmer temperatures simultaneously extended the period of respiration and CO2 loss from wet sedge tundra and heath tundra later into the fall when light limitations prevented primary production (Euskirchen et al. 2017).

In the Antarctic, strong wind events (foehn winds) mobilize dust from exposed mineral surfaces and deposit sediment in topographic depressions of glaciers. Dust and sediment deposited on snow and glaciers reduce the albedo of lower elevation landscapes (Bergstrom et al. 2020) and cause positive feedbacks that drive localized snowmelt and depressions in glaciers (Foreman et al. 2004).

## Conclusions

Drylands play a large role in the Earth's system, and long-term research at US LTER sites is revealing the effects of global warming on these ecosystems. In this article, we provide a novel synthesis from temperate, semiarid, and polar US LTER sites by integrating cross-site observational and experimental data with diverse climate drivers. Although all of the sites exhibited warming, periods of warming and drought differed across US LTER regions and were mediated by multidecadal and interannual climate variability. We demonstrate that climate change affects primary production in dryland US LTER sites in many ways, due to differences in climate, ecosystem characteristics (especially which primary resources are limiting growth), and disturbances among the sites. Interactions among climate drivers, such as heat waves combined with drought, often produced greater responses of primary production, community structure, and ecosystem services than changes in individual climate drivers. Extreme disturbances often produced larger effects than gradual warming. The intensity and frequency of climate-driven disturbance events dictated the length of recovery in ANPP. Projections of more intense and frequent events for more systems on a backdrop of warming will likely cause a slower rate of ANPP recovery but may also spur productive ecosystem reordering. The effects of climate change on dryland primary production and species composition influenced key ecosystem services of air quality and carbon sequestration and highlight the benefits of biologically diverse dryland ecosystems.

## Author Biographical

Amy R. Hudson (amy.hudson@usda.gov), Debra P. C. Peters, and Kerrie Geil are affiliated with the US Department of Agriculture, Agricultural Research Service's Big Data Initiative and SCINet Program for Scientific Computing in Berwyn Heights, Maryland, in the United States. Debra P. C. Peters is also affiliated with the US Department of Agriculture Agricultural Research Service's Jornada Experimental Range in Las Cruces, New Mexico, in the United States. Debra P. C. Peters and Osvaldo Sala are both affiliated with the Jornada Basin LTER Program, at New Mexico State University, in Las Cruces, New Mexico, in the United States. John M. Blair is affiliated with the Division of Biology at Kansas State University in Manhattan, Kansas, in the United States. Daniel L. Childers and Osvaldo Sala are affiliated with the School of Sustainability at Arizona State University, in Tempe, Arizona, in the United States. Peter T. Doran is affiliated with the Department of Geology and Geophysics at Louisiana State University in Baton Rouge, Louisiana, in the United States. Michael Gooseff is affiliated with the Institute of Arctic and Alpine Research at the University of Colorado in Boulder, Colorado, in the United States. Katherine L. Gross and Nick M. Haddad are affiliated with the W. K. Kellogg Biological Station; Katherine L. Gross is also affiliated with the Department of Plant Biology and Nick M. Haddad is affiliated with the Department of Integrative Biology at Michigan State University in Hickory Corners, Michigan, in the United States. Melissa A. Pastore is affiliated with the Rubenstein School of Environment and Natural Resources at the University of Vermont in Burlington, Vermont, in the United States. Jennifer A. Rudgers is affiliated with the Biology Department at the University of New Mexico in Albuquerque, New Mexico, in the United States. Osvaldo Sala is also affiliated with the Global Drylands Center and the School of Life Sciences at Arizona State University in Tempe, Arizona, in the United States. Eric W. Seabloom is affiliated with the Department of Ecology, Evolution, and Behavior at the University of Minnesota in St. Paul, Minnesota, in the United States. Gaius Shaver is affiliated with the Ecosystems Center at the Marine Biological Laboratory in Woods Hole, Massachusetts, in the United States.
